# The role of insulin-like growth factor 2 mRNA binding proteins in female reproductive pathophysiology

**DOI:** 10.1186/s12958-022-00960-z

**Published:** 2022-06-15

**Authors:** Xiao Xu, Hao-Ran Shen, Jia-Rong Zhang, Xue-Lian Li

**Affiliations:** 1 Department of Obstetrics and Gynecology, Zhongshan Hospital, Fudan University, Shanghai, 200032 People’s Republic of China; 2 Obstetrics and Gynecology Hospital, Fudan University, Shanghai, 200011 People’s Republic of China; 3 Shanghai Key Laboratory of Female Reproductive Endocrine Related Diseases, Obstetrics and Gynecology Hospital, Fudan University, Shanghai, 200011 People’s Republic of China

**Keywords:** IMP, Ovary, PCOS, Pregnancy, PE, GDM

## Abstract

Insulin-like growth factor 2 (IGF2) mRNA binding proteins (IMPs) family belongs to a highly conserved family of RNA-binding proteins (RBPs) and is responsible for regulating RNA processing including localization, translation and stability. Mammalian IMPs (IMP1-3) take part in development, metabolism and tumorigenesis, where they are believed to play a major role in cell growth, metabolism, migration and invasion. IMPs have been identified that are expressed in ovary, placenta and embryo. The up-to-date evidence suggest that IMPs are involved in folliculogenesis, oocyte maturation, embryogenesis, implantation, and placentation. The dysregulation of IMPs not only contributes to carcinogenesis but also disturbs the female reproduction, and may participate in the pathogenesis of reproductive diseases and obstetric syndromes, such as polycystic ovary syndrome (PCOS), pre-eclampsia (PE), gestational diabetes mellitus (GDM) and gynecological tumors. In this review, we summarize the role of IMPs in female reproductive pathophysiology, and hope to provide new insights into the identification of potential therapeutic targets.

## Introduction

Insulin-like growth factor 2 (IGF2) mRNA binding proteins (IMPs or IGF2BPs) belong to a highly conserved family of RNA-binding proteins (RBPs), which function to regulate the localization, stability and translation of mRNA and fine-tune the physiological function of the proteins encoded [1]. Members of the IMP family include IMP1–3 in mammals; *Vegetal-1* mRNA-binding protein (Vg1RBP/Vera) in *Xenopus laevis*; zipcode-binding protein 1 (ZBP1) in chickens; murine coding region determinant-binding protein (CRD-BP) in mice; and *Drosophila* IMP (dIMP) [2, 3]. IMP1 is an ortholog of CRD-BP and ZBP1, which are respectively involved in the prevention of c-MYC degradation and the regulation of β-actin mRNA localization [4, 5]. Vg1RBP/Vera, also known as K homology domain-containing protein overexpressed in cancer (KOC), and human IMP3 are also orthologs [6]. Homologs of human IMP2 have been identified as *IGF2BP2a* and *IGF2BP2b* in *Danio rerio*. Moreover, the p62 protein, a 62 kDa isoform generated by IMP2, has been identified as an autoantigen in human hepatocellular carcinoma [7]. Mammalian IMPs (IMP1–3) were originally discovered in studies of embryogenesis [8] and subsequently recognized as oncofetal proteins. IMPs play a critical role in many cancers [9–11] and metabolic diseases [12], due to their role in the regulation of cell processes including cell proliferation, metabolism, invasion and migration. Immunohistochemistry and western blot studies of adult female gonadal tissue report expression of IMP1–3 in the ovary and placenta [13]. Accumulating evidence has shown that IMPs are involved in many aspects of reproductive physiology such as oocyte development [14], ovulation [15], implantation [16] and placentation [17]. Recent studies have implicated the dysregulation of IMPs in polycystic ovary syndrome (PCOS) [18], pre-eclampsia (PE) [19–21] and gestational diabetes mellitus (GDM) [22], in addition to endometrial and ovarian cancers [23, 24]. There are few relevant reports systematically describing the function of IMPs in female reproductive physiology and pathology. Thus, we present the first comprehensive review of the roles of IMPs in female reproductive pathophysiology and provide insights into potential underlying etiological mechanisms of female reproductive disorders.

### Overview of IMPs

The protein structures of the three identified human IMPs (IMP1-3) are highly similar in domain order, and share an overall amino acid sequence identity of 56% [25]. Their canonical structure contains two RNA recognition motifs (RRM) located in the N-terminal region and four nuclear ribonucleoprotein (hnRNP) K homology (KH) domains in the C-terminal region [8]. The RRMs may help to stabilize IMP-mRNA complexes (protein-RNA complexes), and the KH domains mainly contribute to the binding of RNA [26]. Structural analyses indicate that binding of the KH domains 3 and 4 to mRNA induces a conformational change in the transcript, and therefore helps RBPs to assemble higher-order complexes through sequence-specific interactions, providing evidence for their high binding affinity and specificity [8, 27]. The long half-life of IMP-mRNA complexes confers high stability [28]. Moreover, RNA-binding motifs permit IMPs to bind cooperatively with more than one RNA simultaneously, permitting dimerization and formation of stable complexes.

The function of IMPs in the regulation of RNA metabolism has been widely studied, including RNA localization, stability and translation. IMPs are found to be predominantly expressed in the cytoplasm where they form messenger ribonucleoprotein (mRNP) granules around the nucleus which function to transiently “lock” target mRNAs. IMP1 granules have been shown to be particularly prevalent in neurons and oocytes. Compositional analysis has identified the exon-junction components, CBP80 and poly(A)-binding protein in IMP1-containing mRNP granules; the presence of which represents pre-translational status. IMP1 has also been shown to mediate transport of *β-actin* mRNA to the leading edge of motile cells.

IMPs were originally discovered as mRNA-binding proteins capable of binding to the 5’ untranslated regions (5’UTR) of IGF2 [8]. The *IGF2* gene generates numerous mRNAs with different 5’UTRs. The human *IGF-2* gene is a complex transcription unit driven by four promoters. Each promoter directs transcription of a different RNA designated leader. Four designated leaders of IGF2 have different translational properties. IMP1-3 have been shown to regulate the translation of IGF2 through binding with the IGF2 mRNA designated leader 3 rather than IGF2 mRNA designated leader 4 in a rapamycin-sensitive manner. The co-translational phosphorylation of IMP1 is catalyzed by mTOR complex 2, which mediates initiation of IGF2-L3-luciferase mRNA translation by cap-independent internal ribosomal entry [29]. IMP2 may also be phosphorylated by the mTOR complex 1 to promote translation of IGF2 mRNA by internal ribosomal entry [30]. Knockout of IMP3 results in the inhibition of translation of IGF2 and reduction in the levels of both intracellular and secreted IGF2 [31]. The IMP-IGF2 pathway has been implicated in the pathogenesis of many diseases. For example in immune thrombocytopenia, inactivation of IMP1-IGF2 signaling, caused by overexpression of miR-98-5p, has been shown to repress the phosphatidylinositol 3-kinase (PI3K)/Akt pathway and play a role in the deficiency of mesenchymal stem cells [32]. In cases of hypoxic-ischemic brain injury, activation of IMP2-IGF2 signaling, mediated by RNA-binding motif protein 3, promotes neural stem cell and progenitor cell proliferation and differentiation in the sub-granular zone [33]. In chicken myoblasts, the inhibition of IMP3-IGF2 pathway elicited by let-7b repressed cell proliferation has been found to be involved in the pathogenesis of dwarfism. In the female reproductive system, IGF2 has been universally found in follicular fluid [34], granulosa cells (GC) [35] and theca cells [36], and plays a role in folliculogenesis [37] and embryogenesis [38]. IGF2 is mainly secreted from granulosa cells, and collaborates with IGF1 to stimulate steroid hormone synthesis and promote proliferation of GCs through acting on IGF1R [39]. IMPs may therefore contribute to the normal physiology and pathological conditions of the female reproductive system through the regulation of IGF2. Other mechanisms of action of IMPs in the female reproductive system are described below.

### Distribution of IMPs in the ovary

Expression of all three IMPs have been identified in adult ovarian and placental tissue. The physiological expression of IMP1–3 exhibits a biphasic pattern during development. IMP1–3 first appears in the oocyte and persists from pre-fertilization to the stages of zygote and blastocyst during early embryogenesis, and is then found to decline until murine embryonic day 10.5–12.5, when expression increases again [13]. In the adult human ovary, immunohistochemical studies indicate that IMPs are expressed in the cytoplasm of resting and growing oocytes, as well as GCs of small and growing follicles, with strong immunostaining of IMP2 and weak immunostaining of IMP1and IMP3. In human fetal ovaries, strong immunostaining of IMP3 and weak immunostaining of IMP1 were restricted to developing follicles from 32 weeks gestation, while IMP2 immunostaining was ubiquitous. Evidence from immunohistochemical analysis suggests that IMP1–3 may therefore play several different roles in folliculogenesis.

### IMPs in embryogenesis

After fertilization, the early embryo undergoes maternal-to-zygotic transition (MTZ), which is the stage during which regulation of development, previously controlled by maternal factors, changes to be under the control of new genetic products from the zygotic genome. The transition includes two distinct processes: first, the maternal information (mRNAs and proteins) is gradually cleared; second, zygotic transcription is activated [40]. Many maternal factors are present from oocyte to embryo and directly regulate early development in vertebrates, such as egg activation, embryonic cell division, and cytoskeleton assembly [41–43]. Maternally inherited mRNAs are stable during the first few hours of embryonic development, and then subsequently degrade [40]. During the MTZ, initiation of zygotic transcription, known as zygotic genome activation (ZGA), is mediated by maternally derived factors [44]. Although the exact timing of ZGA during MTZ remains uncertain, some genes involved in the process have been identified. IMP3 acts as an RNA stabilizer during early development, and depletion of maternal IMP3 destabilizes maternal mRNAs prior to MTZ and leads to severe developmental defects, including abnormal organization of the cytoskeleton and aberrant cell division [45]. Moreover, overexpression of IMP3 enhances the stability of its target maternal mRNAs, decelerates clearance of maternal mRNA, and inhibits MTZ, resulting in developmental delay [45, 46]. Thus, maintaining a balance of IMP3 is essential for normal early development. Deletion of IMP2 has been shown to cause early embryonic developmental arrest by targeting the 3’UTR of CCAR1 and RPS14 during ZGA [38]. IMP2 also enhances IGF2 mRNA stability and promotes IGF2 translation. In vitro experimentation suggests that supplementation of culture media of derived mouse embryos with IGF2 remarkably improves the proportion of embryos that successfully develop compared with a control group (IMP2^♀+/♂+^), but exerts no effect on IMP2^−/−^female-derived embryos (IMP2^♀−/♂+^). This effect of IGF2 has also been observed in human embryos [38]. The data suggest that IMP2 is involved in ZGA in part by regulating the stability and translation of IGF2.

Embryo implantation is a dynamic and complex reproductive process regulated by a series of molecular and cellular events with its ultimate success based on the established uterine receptivity [47, 48]. Both abnormal hereditary and epigenetic modifications can affect uterine receptivity and therefore disturb embryo implantation and result in spontaneous abortion [49, 50]. Whole-genome bisulphite sequencing and differentially methylated region (DMR) analysis showed that 21,391 DMRs were found to be hypomethylated in the receptive endometrium of goats on day 15 of gestation compared with the pre-receptive endometrium on day 5 of gestation. The methylation ratio of IMP2 has been found to be lower in the receptive endometrium of goats than in the pre-receptive endometrium, while methylation ratios of IMP3 and IGF1R genes are higher [33], which may be a compensatory response. Abnormal expression of IMPs in endometrium may affect embryo implantation by affecting uterine receptivity. Many members of the let-7 family have been found to inhibit IMP expression by binding to the 3’UTR of IMP mRNA in various cell types. IMP1 has been identified as a target of let-7a [51, 52], let-7b [53], let-7f [54] and let-7i [55]. IMP2 has been identified as a target of let-7a [56], let-7b [57, 58] and let-7i [55] and IMP3 has been identified as a target of let-7b [59] and let-7i [55]. Interestingly, the expression of members of the let-7 family has been found to be lower in endometrial samples with endometritis than in clinically healthy endometrium, in particular let-7e and let-7f that are repressed in both subclinical and clinical endometritis, which may disturb uterine homeostasis and affect uterine receptivity [58]. Therefore, IMPs may potentially play a role in establishing endometrial receptivity, mediated by members of the let-7 family.

**Fig. 1:**
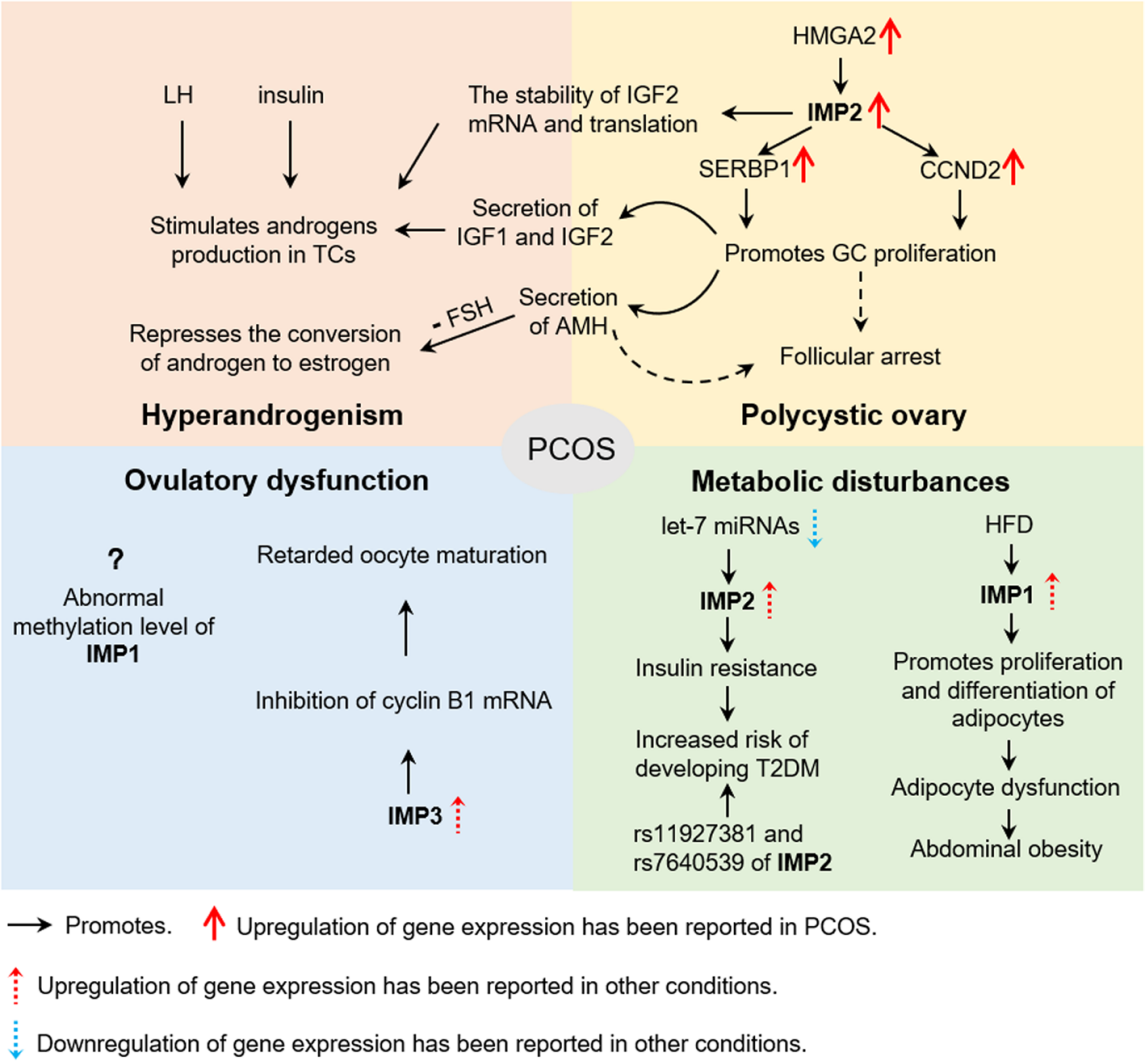
Abnormal expression of IMPs in the pathogenesis of PCOS

Early placentation is critical to both perinatal fetal growth and postnatal fetal and maternal health. Transcriptome analysis performed during the implantation period revealed that there is overexpression of some placental growth factors, and IMP1 and IMP3 in human trophoblast ectoderm cells, as well as upregulation of corresponding receptors in the receptive endometrium [16], implying that these molecules play an important role in the early dialogue between the blastocyst and maternal endometrial cells. Cytotrophoblast cells are found to proliferate and differentiate into several different trophoblast lineages during early gestation [60] and dysregulated proliferation and differentiation of cytotrophoblast cells can result in severe developmental disorders including intrauterine growth retardation and perinatal death [61, 62]. In sheep, rapid proliferation of trophoblast cells occurs in the process of conceptus elongation, which is essential for the implantation, placentation, and successfully establishing pregnancy [63, 64]. Knockout of LIN28 (LIN28A and LIN28B) and the resultant increase in let-7 micro-RNAs (miRNAs) in day 16 trophectoderm cells have been shown to reduce the degree of elongation of the conceptus and down-regulate the expression of IMP1, IMP2, IMP3, HMGA1, ARID3B, and c-MYC. This results in impaired placentation, fetal growth restriction and reduced fertility in sheep [17]. Therefore it is clear that IMPs play a major role in many developmental processes including ZGA, early embryonic development, implantation and placentation (Fig. 1), but the specific regulatory mechanisms employed in humans require further exploration.

In mouse parthenogenetic embryos, the expression of IMP1 and IMP2 is perturbed compared to that in embryos which have been normally fertilized. As N^6^-methyladenosine (m^6^A) readers, IMPs recognize m^6^A modifications, which regulate splicing, translocation, stability, and translation. In mammals, the occurrence of abnormal m^6^A modifications during early development results in parthenogenetically activated oocytes that are not capable of developing to term [65]. Reduced expression of IMP1 elicited by miR-670 decreases the rates of cleavage and blastula formation in parthenogenetically activated embryos via the down-regulation of m^6^A expression [66]. This effect can be reversed by treatment with betaine or miR-670 inhibitor.

### IMPs and female reproductive pathologies

#### IMPs and PCOS

Polycystic ovary syndrome (PCOS) is a common disorder of the ovary in women of reproductive age, and is characterized by hyperandrogenism, ovulatory dysfunction (anovulation or oligo-ovulation) and a polycystic ovarian morphology [67]. Most PCOS patients also suffer from metabolic abnormalities, such as insulin resistance and abdominal obesity which suggests potential adipocyte dysfunction, and these patients also have an increased lifetime risk of developing type 2 diabetes mellitus (T2DM) [68]. Multiple pathologic mechanisms have been found, however the exact etiology of PCOS is not fully understood.

In polycystic ovaries, early follicular growth is excessive, resulting in massive ovarian preantral follicles, which become arrested and do not proceed to develop a dominant follicle. PCOS is also usually accompanied by high rates of GC proliferation and low rates of GC apoptosis. GC is an important source of anti-Müllerian hormone (AMH), IGF-1 and IGF-2 in the ovary. Elevated AMH has an antagonistic effect against the action of follicle stimulating hormone (FSH) on GCs, and represses the conversion of androgens to estrogens, therefore aggravating the androgen excess found in PCOS. Large numbers of GCs associated with excessive amounts of small ovarian follicles, which have become arrested in part, account for the characteristic polycystic morphology of the ovaries in PCOS. The expression of IMPs in PCOS patients is controversial and IMP2 may play a more important regulatory role in ovaries than IMP1 and IMP3. A recent microarray analysis identified that IGF1R, IGF2R, IMP2, IGFBP2 and IGFBP7 were all found to be down-regulated in the cumulus cells from patients with PCOS compared with patients without PCOS [69]. In contrast, a recent study showed elevation of expression of IMP2 in GCs and ovarian tissue of PCOS patients compared with that of a control group [18]. This contradiction may be due to the small sample size of the earlier study. A genome-wide association study (GWAS) showed that the high mobility group AT hook 2 (HMGA2) gene may be a high risk gene for PCOS and the expression of HMGA2 in ovarian tissue and ovarian GCs of PCOS patients has been shown to be higher than that in a control group. IMP2 can be upregulated by overexpression of HMGA2 and can promote GC proliferation by targeting the 3’UTR of *cyclin D2 (CCND2)* and *SERPINE1 mRNA binding protein 1 (SERBP1)* mRNA [18]. In the ovary, IGF2, IGF1 and insulin are capable of synergistically stimulating androgen secretion from theca cells [70]. Abnormally elevated expression of IMP2 promotes the translation and stability of IGF2. Increased secretion of IGF2 stimulates the production of androgens from theca cells in ways that are synergistic with IGF1 and insulin, which helps to further explain the androgen excess found in PCOS patients.

Ovulatory dysfunction may be partially due to impaired oocyte maturation. In the zebrafish oocyte, IMP3 has been found to retard the progression of oocyte maturation by repressing translation of *cyclin B1* mRNA [71]. Upregulation of IMP3 in bovine oocytes is related to a lower vitrification temperature, which may affect stress prevention and oocyte recovery [72], however temperature-dependent regulation of IMP3 in human oocytes remains unknown.

**Fig. 2:**
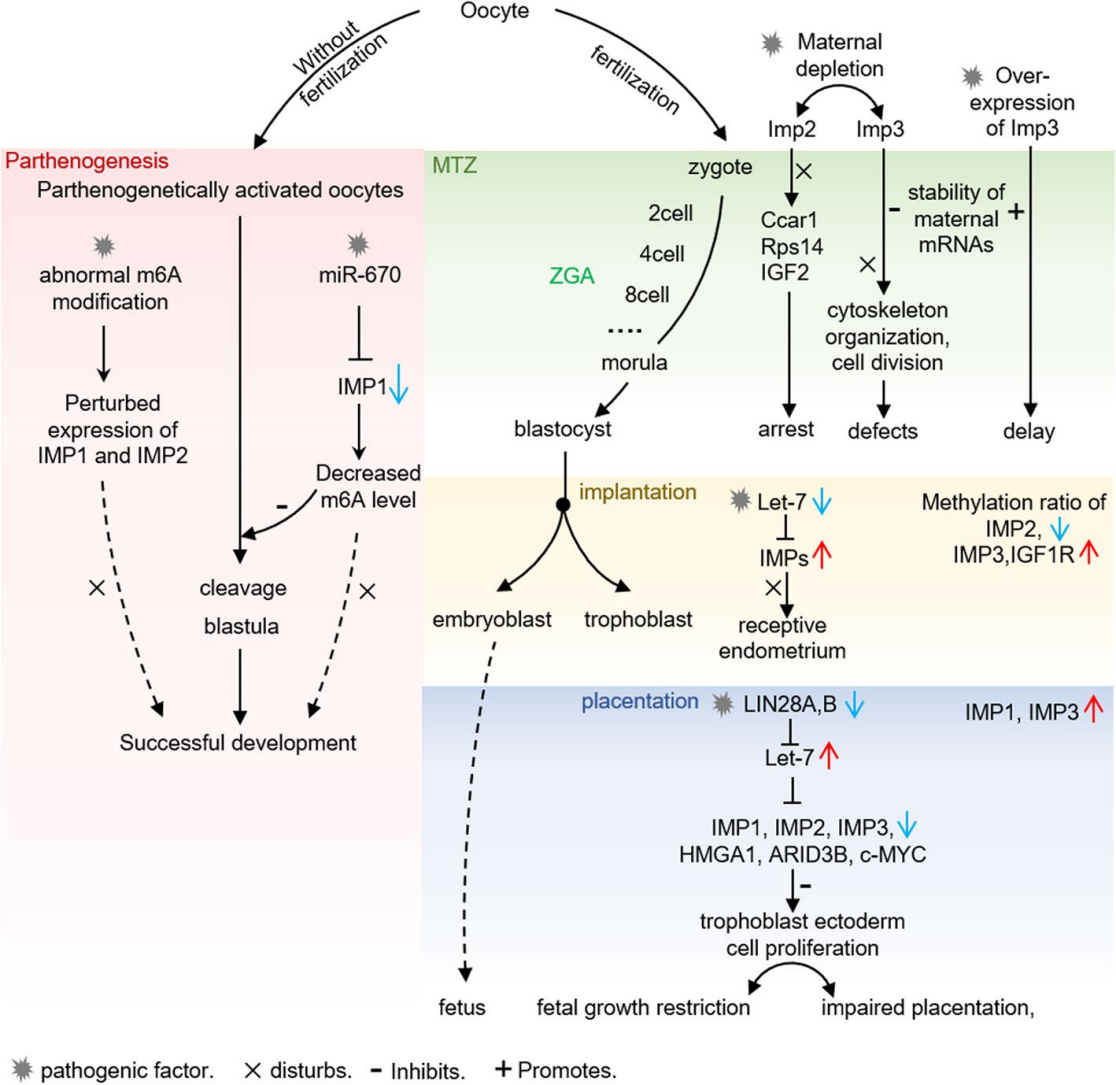
The effect of IMPs on parthenogenetic activation, zygotic genomic activation, early embryonic development, implantation and placentation

As mentioned earlier, many patients with PCOS have other concurrent metabolic disorders, such as insulin resistance, hyperinsulinemia, dyslipidemia and activation of proinflammatory factors, and these patients also have a long term risk of developing T2DM and obesity [68, 73, 74]. A case–control study suggested that variants of T2DM risk genes are significantly more frequently found in patients with PCOS than in healthy controls [75]. There is a transcription-regulatory mechanism involved in regulating insulin secretion and glucose metabolism. Conditional inactivation of IMP2 has been shown to result in impaired insulin secretion from the islet cells of mice [76]. Reduced expression of multiple miRNAs including let-7b-5p, miR-1-3p, miR-24-3p, miR-34a-5p, miR-98-5p, and miR-133a-3p, has been observed in the peripheral blood of T2DM patients compared with controls and expression levels appear to correlate with serum insulin levels, HbA1c levels and body mass index (BMI) [77]. These miRNAs target susceptibility genes for T2DM including CDKN2A, CDK5, IMP2, KCNQ1, and TSPAN8. These results suggest that the elevated expression of IMP2 observed in PCOS may also play a role in inducing insulin resistance in addition to the abnormal promotion of GC proliferation. The molecular mechanisms of IMP2 related to the regulation of metabolism in PCOS remain to be confirmed. IMP1 is a critical regulator of fat metabolism and adipogenesis, but whether IMP1 influences the metabolic abnormalities found in PCOS patients remains to be explored. Interestingly, a study reported that metformin was found to downregulate H19 via mediation of DNA methylation [78]. H19 is a long noncoding RNA, which harbors multiple let-7 binding sites and acts as a ‘sponge’ to prevent let-7 from inhibiting expression of target genes [79, 80]. The data suggest that metformin may repress IMPs by downregulating the H19/let-7 pathway of DNA methylation, which may be one of its therapeutic mechanisms. All in all, the antagonistic action of miRNAs and IMPs, as well as the regulation of IMPs on target genes are both essential for the physiological and pathological processes involved in PCOS. IMPs are likely to participate in the pathogenesis of PCOS in a variety of ways (Fig. 2).

#### IMPs and PE

Abnormal proliferation and differentiation of human placental trophoblasts result in abnormal trophoblast invasion and dysfunctional syncytialization, which are involved in the pathogenesis of PE [81]. As a serious pregnancy-related disease manifesting with hypertension and proteinuria, PE is one of the main conditions contributing to maternal mortality, perinatal death, preterm birth and intrauterine growth retardation [82]. The expression of miR-423-5p is increased in the placentas of pre-eclamptic patients and overexpression of miR-423-5p has been found to inhibit migration, invasion and proliferation as well as inducing apoptosis in HTR-8/SVneo cells via targeting IMP1 [21]. Elevated expression level of miR-181a-5p in both plasma and placenta of severe pre-eclamptic patients suppresses invasion and migration of HTR-8/SVneo cells by directly inhibiting IMP2 [20]. During normal pregnancy, IMP3 is highly expressed in human placental villi in the first trimester compared with the third trimester, particularly in cytotrophoblast cells and column trophoblast cells [19], therefore migration and invasion of trophoblast cells from the placenta in the first trimester are promoted compared with the third trimester [83]. Silencing of IMP3 notably represses the invasion and migration of first trimester human placental villi and decreases the mRNA level of IGF2 and CD44 in HTR8/SVneo cells [19]. IGF2 is reported to activate and stimulate the PI3K/AKT pathway and promote the migration of ovine trophoblast ectoderm cells [84, 85]. In addition, the phosphorylation of AKT promotes the metastasis of multiple tumor cells. Lower expression of IMP3 has been observed in the placentas of PE patients compared with placentas from healthy women [19]. Therefore, these studies suggest that down-regulation of IMP3 mediates abnormal invasion and migration of placental trophoblast cells, probably via IGF2-PI3K-AKT pathways, thus contributing to the development of PE.

#### IMPs and gestational diabetes mellitus (GDM)

Meta-analyses of candidate gene studies and GWAS have identified multiple genes which are reproducibly associated with GDM, including TCF7L2, GCK, KCNJ11, KCNQ1, CDKAL1, IMP2, MTNR1B, and IRS1. These genes are also associated with T2DM [86]. Among pregnant women, the presence of polymorphisms in IMP2 has been shown to be highly correlated with the occurrence of GDM [87]. Rs4402960 (IMP2) and rs1801278 (Gly972Arg, IRS1) have been shown to be significantly associated with a higher risk of GDM [88–90].

#### IMPs and gestational trophoblastic diseases

Gestational trophoblastic disease is caused by excess cellular proliferation of placental villous trophoblast cells, and includes a spectrum of cellular abnormalities including hydatidiform mole (HM), invasive mole, choriocarcinoma, and placental site trophoblast tumor. HMs can be divided into complete, partial and invasive HMs [91]. According to histopathological features, partial HMs appear to be similar to normal placenta, while complete and invasive HMs are more like choriocarcinoma with multiple abnormally expressed oncogenes [92]. Most previous studies have shown that high expression of IMP3 is positively correlated with the occurrence or progression of cancer. Conversely, IMP3 is strongly expressed in extravillous cytotrophoblasts in healthy placenta, with expression level gradually decreasing from partial HMs, to complete HMs to invasive HMs [93]. The data suggest that IMP3 expression is negatively correlated with the malignant potential of HMs, but the direct mechanisms require further exploration. In the study of choriocarcinoma, β-Catenin, as a critical mediator of the Wnt signaling pathway, promotes the proliferation of human choriocarcinoma cells via the LIN28B/Let-7a/IMP1 pathway which mediates inhibition of let-7a, promotes IMP1 expression and thereby promotes proliferation of JAR cells. High expression of LIN28B has been observed in choriocarcinoma tissue compared with normal placental villi. However, whether the expression of IMP1 in choriocarcinoma promotes cell proliferation needs to be further explored in clinical specimens [52].

#### IMPs and gynecological tumors

Overexpression of IMP3 is associated with an unfavorable overall survival of patients with epithelial ovarian carcinoma (EOC) [94], such as ovarian clear cell carcinoma (OCCC) [23, 95], ovarian serous carcinoma [96] and primary ovarian mucinous carcinoma [97]. Knockout of IMP3 inhibits cell proliferation, migration and invasion, and downregulates the translation of MMP-2 and MMP-9 in OCCC cells [98]. Elevated IMP1 levels have been observed in ovarian carcinoma compared with normal peritoneum [99]. IMP1 stabilizes c-MYC and β-TrCP1 MRNA transcripts and has been shown to promote cell proliferation in IGROV-1 ovarian carcinoma [100]. IMP1 enhances the phenotype of invasive tumor cells by reducing the down-regulation of its miRNA-regulated target MRNA [101]. The effect of IMP1 on cancer-derived cells is conserved, while the roles of IMP2 and IMP3 vary in a cell-dependent manner. Immunohistochemistry has revealed strong staining of IMP3 and IMP2 in both ovarian serous carcinoma and tubal cancers and therefore IMP3 and IMP2 may be useful as biomarkers of pelvic high-grade serous carcinoma [102, 103].

Uterine leiomyomas (ULM) is characterized by histological and molecular heterogeneity. The pathogenesis of ULM is partially a result of several driver gene mutations, such as MED12 mutation, HMGA2 overexpression, and biallelic FH inactivation [104, 105]. ULM with HMGA2 overexpression also has increased IMP2 expression, as well as higher levels of AKT signaling and mitogenic activity than other ULM types. HMGA2 has been shown to activate AKT signaling through upregulation of IMP2 in embryonic rhabdomyosarcoma [106]. Knockout of HMGA2 in ULM cells causes inactivation of AKT signaling and upregulation of p16 and p21, which eventually lead to cell arrest [107]. In uterine leiomyosarcoma, IMP3 is an independent marker of poor prognosis [108]. In addition, IMP3 was upregulated in HPV16-positive cervical cancer and precancerous tissues compared with normal tissues and facilitated aerobic glycolysis by stabilizing HK2 mRNA, consequently promoting the malignant phenotype in cervical cancer cells [109].

Endometrial cancer is a common gynecologic malignancy, which originates from the endometrium and is composed of heterogeneous populations of cells. Traditionally, it has been classified into two types, type I (estrogen-dependent) and type II (estrogen-independent) based on clinical and epidemiological features [110]. Pathologically, endometrioid carcinoma is the most common subtype of type I endometrial carcinoma, and type II generally includes serous carcinoma and clear cell carcinoma [111]. On the basis of immunohistochemistry, the key biomarkers that are useful in the differentiation of endometrioid and serous endometrial carcinomas are estrogen receptors, progesterone receptors, IMP3, p53, and p16 [52, 112].

## Conclusions

In summary, IMPs are implicated in the physiology and pathology of the female reproductive system. As a biomarker, IMPs promise to be very useful in being able to monitor the healthy embryogenesis and oocyte maturation, and to predict the occurrence and progression of some female reproductive diseases (Table 1). The expression of IMP2 is increased in ovarian GCs of women with PCOS, while the expression of IMP1, IMP2 and IMP3 is decreased in the placental tissue of women with PE. There is an association between down-regulation of IMPs and some adverse pregnancy outcomes, however the exact mechanisms are not yet fully understood. Although the role of the IMP family in tumorigenesis has been extensively explored, the role in diseases of the female reproductive system is still under investigation, with many studies conducted in other species and few in human tissue. Study limitations including small sample sizes, inadequate measurement methods and lack of control for confounding factors preclude our ability to draw firm conclusions from the current evidence. More focused studies are needed to address these limitations in order to disentangle the functions of IMPs and to clarify the clinical value as a novel biomarker of female reproductive diseases.

**Table 1:** The role of IMPs in the embryogenesis, oocyte maturation, and female reproductive pathology

Condition		Cell type/Species	Key findings	Proposed mediator	Implications	References
Embryogenesis	MTZ	Zebrafish oocyte	IMP3 ↓ leads to abnormal cytoskeleton organization and cell division	Maternal mRNAs	IMP3 depletion leads to severe developmental defects	[45]
		Zebrafish oocyte	IMP3 ↑ inhibits maternal-to-zygotic transition	Maternal mRNAs	Leads to developmental delay	[46]
	ZGA	Mice	IMP2 ↓causes early embryonic developmental arrest	Ccar1, Rps14, IGF2	Abundant IMP2 is essential for the embryo development	[38]
	Implantation	Human/Bovine	Methylation ratio of IMP2↓and IMP3 ↑in receptive endometrium	No data	Predicts the estrous phase	[49, 50]
	Placentation	Human	IMP1 and IMP3 ↑	Corresponding receptors	Early dialogue between blastocysts and maternal endometrial cells	[16]
		Ovine	IMP1-3↓	LIN28, let-7	Increases the degree of elongation of the conceptus abnormal m6A modification	[64]
	PA	Mice	The expression of IMP1 and IMP2 is perturbed	No data	during early development in PA embryos	[65]
		Mice	IMP1↓	miR-670	Regulates RNA methylation in PA mouse embryonic development	[66]
Oocyte maturation		Zebrafish oocyte	IMP3 retards the progression of oocyte maturation	Cyclin B1	Represses oocyte maturation	[71]
PCOS		Human	IMP2↓in cumulus cells from PCOS patients	No data	Predictive biomarker	[69]
		Human	IMP2↑in GCs and ovary	CCND2, SERBP1	Provides new insights into the dysfunction of GCs in PCOS	[18]
PE		HTR-8/Svneo cells	IMP1 inhibits apoptosis in HTR-8/Svneo cells	miR-423-5p	A new light on the pathogenesis of severe pre-eclampsia	[21]
		HTR-8/Svneo cells	IMP2 promotes the invasion and migration of HTR-8/Svneo cell	miR-181a-5p	The roles and molecular mechanisms of IMP2 in PE	[20]
		Human placenta	IMP3 ↓in the placentas from PE patients	IGF2, CD44	Predictive biomarker	[19]
GDM		Human	Rs4402960 (IMP2)	No data	Associated with a higher risk of GDM	[86–88]
Gynecological oncology	ULM/ULMS	Human	IMP2/IMP3	No data	Predictive marker/independent prognostic factor	[105, 108]
	Cervical carcinoma	SiHa cells	IMP3 ↑ facilitate cell aerobic glycolysis	Hexokinase 2	A potential approach for cervical cancer therapeutics	[109]
	Choriocarcinoma	JAR cells	IMP1 ↑ promotes cell migration and invasion	RSK2, PPME2	Aggravating the choriocarcinoma disease progression	[52]
	Endometrial carcinoma	Human	IMP3 ↑, PR, L1CAM	No data	Histological subtyping in high-grade EECs	[112]
	Ovarian carcinoma	Human	IMP1, IMP2, IMP3 and E-cadherin	No data	Predicts disease recurrence and survival	[97–99]

*Abbreviation*: *MTZ* Maternal-to-zygotic transition, *ZGA* Zygotic genome activation, *PA* Parthenogenetic activation, *PCOS* Polycystic ovary syndrome, *PE* Pre-eclampsia, *GDM* Gestational diabetes mellitus, *ULM* Uterine leiomyomas, *ULMS* Uterine leiomyomasarcoma

## Data Availability

Not applicable.
